# NHLRC2 expression is increased in idiopathic pulmonary fibrosis

**DOI:** 10.1186/s12931-022-02129-z

**Published:** 2022-08-13

**Authors:** Mervi Kreus, Siri Lehtonen, Reetta Hinttala, Johanna Salonen, Katja Porvari, Riitta Kaarteenaho

**Affiliations:** 1 Research Unit of Internal Medicine, University of Oulu, Oulu, Finland; 2 Center of Internal Medicine and Respiratory Medicine and Medical Research Center Oulu, Oulu University Hospital, Oulu, Finland; 3 Department of Obstetrics and Gynecology and Medical Research Center Oulu, Oulu University Hospital, Oulu, Finland; 4 Medical Research Center Oulu and PEDEGO Research Unit, University of Oulu and Oulu University Hospital, Oulu, Finland; 5 Biocenter Oulu, University of Oulu, Oulu, Finland; 6 Department of Forensic Medicine, University of Oulu, Oulu, Finland

**Keywords:** Idiopathic pulmonary fibrosis, Fibroblast focus, Hyperplastic alveolar epithelium, Smoking, Acute exacerbation

## Abstract

**Background:**

Variants of NHL repeat-containing protein 2 (NHLRC2) have been associated with severe fibrotic interstitial lung disease in early childhood and *NHLRC2* has been listed as a differentially expressed gene between rapidly and slowly progressing idiopathic pulmonary fibrosis (IPF) patients. However, its cell type-specific localization in human lung tissue is unknown. The aim of this study was to evaluate NHLRC2 mRNA and protein expression in different cell types of lung tissue samples and to investigate the effect of transforming growth factor (TGF)-β1 exposure on NHLRC2 expression in vitro.

**Methods:**

The NHLRC2 expression in lung tissue samples was studied by immunohistochemistry (50 IPF, 10 controls) and mRNA in situ hybridization (8 IPF, 3 controls). The immunohistochemical NHLRC2 expression was quantified with image analysis software and associated with the clinical and smoking data of the patients. NHLRC2 expression levels in primary stromal and small airway epithelial cell lines after exposure to TGF-β1 was measured by quantitative reverse transcription polymerase chain reaction and Western blot analysis.

**Results:**

NHLRC2 expression was detected especially in bronchiolar epithelial cells, type II pneumocytes and macrophages in normal lung. In the lungs of IPF patients, NHLRC2 was mainly expressed in hyperplastic alveolar epithelial cells lining fibroblast foci and honeycombs. NHLRC2 expression assessed by image analysis was higher in IPF compared to controls (p < 0.001). Ever-smokers had more prominent NHLRC2 staining than non-smokers (p = 0.037) among IPF patients. TGF-β1 exposure did not influence NHLRC2 levels in lung cell lines.

**Conclusions:**

NHLRC2 expression was higher in IPF compared to controls being widely expressed in type II pneumocytes, macrophages, bronchiolar epithelium, and hyperplastic alveolar epithelium. Additionally, its expression was not regulated by the exposure to TGF-β1 in vitro. Further studies are needed to clarify the role of NHLRC2 in IPF.

**Supplementary Information:**

The online version contains supplementary material available at 10.1186/s12931-022-02129-z.

## Background

Idiopathic pulmonary fibrosis (IPF), a chronic and progressive interstitial lung disease, is considered to result from a failure of alveolar epithelial cell repair after repetitive injury leading to increased expression of profibrotic mediators, including transforming growth factor (TGF)-β1, and activation of fibroblasts and abnormal wound healing responses [1, 2]. An unpredictable clinical course of disease is typical for IPF. Some IPF patients experience acute exacerbations (AE), defined as an acute, clinically significant respiratory deterioration [3].

The histological pattern of IPF, referred to as usual interstitial pneumonia (UIP), is characterised by heterogeneous lesions with dense fibrosis, fibroblast foci (FF) consisting of fibroblasts and myofibroblasts, metaplastic and hyperplastic changes in epithelial cells lining the alveoli and re-epithelialized air spaces i.e., honeycomb cysts [4]. The most common histopathological finding of AE-IPF is diffuse alveolar damage (DAD) superimposed on underlying UIP [5]. Single-cell RNA sequencing studies of IPF lung tissue have provided evidence for new epithelial cell type termed as an aberrant basaloid cell that express markers of both alveolar epithelial cells and basal cells [6]. Serum levels of certain epithelial cell markers, including matrix metalloproteinase 7 and cancer antigen 125 (also known as mucin 16) are identified being indicative of the presence, severity, and prognosis of IPF [7–9].

Certain variants of NHL repeat-containing protein 2 (NHLRC2, gene name *NHLRC2*) have been reported to cause multi-organ disease with severe fibrotic interstitial lung disease in early childhood (OMIM #618278) [10–12]. One gene expression study has previously listed *NHLRC2* as a down-regulated gene when comparing surgical lung tissue samples of rapidly progressing (the percent predicted forced vital capacity (FVC%) and diffusing capacity of carbon monoxide (DLCO%) declined significantly up to 12 months following biopsy) to relatively stable IPF patients [13]. High *NHLRC2* gene expression in lung tumour samples has been associated with a long survival time in lung adenocarcinoma patients in the study utilizing three datasets from Gene expression omnibus database [14]. Up-regulated *NHLRC2* levels in lung tissues of heaves-affected horses have been reported after an antigen challenge in one gene expression study [15]. NHLRC2 cell type-specific localization, however, in normal or diseased human lung tissues has not been previously studied.

This study aimed to examine the cell type-specific expression of NHLRC2 protein and mRNA in lung tissue of patients with IPF by immunohistochemistry (IHC) and in situ hybridization. The results of lung tissue samples analysed by IHC and digital pathology image analysis were associated with the clinical and smoking data of the patients. Protein and mRNA levels of NHLRC2 were also assessed in primary stromal cell lines cultured from patients with IPF and controls as well as respiratory epithelial cell lines. In addition, the effect of TGF-β1 on NHLRC2 gene and protein expression levels in stromal and epithelial cell line cells were measured with quantitative reverse transcriptase polymerase chain reaction (RT-qPCR) and Western blot analysis, respectively.

## Materials and methods

### Patients

The study material used for IHC consists of lung tissues from 50 IPF patients who had undergone surgical lung biopsy at the Oulu University Hospital for diagnostic purposes between 1991 and 2019 as described in the previous study [16]. Lung tissue specimens taken at autopsy from 8 out of 50 patients were also studied. IPF was diagnosed according to the international guidelines [4]. The patients experiencing AE-IPF during follow-up (n = 22) were identified either based on the surgical lung biopsy and autopsy material showing DAD in parallel with UIP indicating AE-IPF (n = 9), or by applying the current criteria for AE-IPF (n = 13) [3]. Control samples (n = 10) were derived from histologically normal-looking lung tissues from non-smoking patients being operated for lung adenocarcinoma.

Date of birth, gender, age, smoking status, pharmacological treatment, and pulmonary function test results at the time of biopsy were collected from electronic patient records. Patients with less than 5 pack-years of smoking history were regarded as non-smokers. The overall survival time was calculated from biopsy date to death, transplantation, or last follow-up date (May 11, 2021). Death dates were collected from death certificates obtained from the national registry of Statistics Finland.

### Immunohistochemistry

Immunohistochemical stainings were performed in serial sections for lung tissue samples from IPF and control patients. Formalin-fixed and paraffin-embedded 3.5 μm thick tissue sections were stained by Envision+ System Kit (Dako, Glostrup, Denmark) with 3,3′-diaminobenzidine chromogen as described previously [17]. Antibodies are listed in Additional file 1: Table S1. NHLRC2 expression was compared to collagen α1(IV) chain (gene name *COL4A1*) based on the results of our previous study on the microarray analysis of lung stromal cells [17]. In order to identify the phenotype of the cells expressing NHLRC2, few cases were also studied for alpha smooth muscle actin (α-SMA, marker for myofibroblasts, gene name *ACTA2*), cluster of differentiation (CD) 68 (marker for macrophages), thyroid transcription factor (TTF)-1, marker for type II pneumocytes) and CD31 (marker for endothelial cells). Rabbit isotype control (Invitrogen, Carlsbad, USA) was used as negative control.

Whole slide images were acquired with a Leica-Aperio AT2 (Leica Biosystems, Nussloch, Germany) in Biobank Borealis of Northern Finland, Oulu University Hospital or with a NanoZoom S60 scanner (Hamamatsu, Hamamatsu city, Japan) in Transgenic and Tissue Phenotyping core facility, Biocenter Oulu, University of Oulu at 40× magnification.

### Digital image analysis of immunohistochemical NHLRC2 expression

Visiopharm image analysis software (Visiopharm Integrator System, Hoersholm, Denmark) provided by Transgenic and Tissue Phenotyping core facility, Biocenter Oulu, University of Oulu was used to determine the area of NHLRC2-positive staining in all types of lung cells considering all intensities (strong, middle, weak, very weak) in relation to total area of the tissue section in 50 surgical lung biopsy samples from IPF patients with a histology of UIP (n = 47) or UIP and DAD (n = 3) and 10 control lung tissue samples.

### Calculation of NHLRC2-positive fibroblast foci

Digitized lung tissue specimens were examined by using Aperio Image Scope (Version 12.4.3.5008, Leica Biosystems) or NDP.view2 (Hamamatsu, Hamamatsu city, Japan). The total number of NHLRC2-positive FF were calculated from 47 samples with UIP histology. NHLRC2-positive FF was determined as consisting of more than 50% of positive stromal cells considering all intensities. In addition, the total number of FF were calculated from the 47 sections with UIP histology. The number of FF was presented in relation to the area of the tissue section.

### mRNA in situ hybridization

*NHLRC2* mRNA in situ hybridization was performed for surgical lung biopsy samples of 8 IPF patients with a histology of UIP (n = 6) or UIP and DAD (n = 2) and in 3 control lung tissue samples using RNAscope 2.5 HD assay—RED and probe Hs-NHLRC2 (555721) according to the manufacturer’s instructions (Advanced cell diagnostics, ACD, Newark, CA, USA). Formalin-fixed and paraffin-embedded specimens were cut into 4 µm thick sections. Target retrieval was performed by boiling the sections at 98 °C for 15 min in RNAscope target retrieval reagent using a KOS Microwave HistoSTATION (Milestone, Sorisole, Italy). Gill’s Hematoxylin (Sigma-Aldrich, St. Louis, MO, USA) was used to stain the nuclei, and coverslips were mounted with EcoMount (Biocare Medical, Pacheco, CA, USA). Positive and negative control probes (Hs-UBC 310041 and DapB 310043, ACD) were used to help qualify samples and control for background noise. Specific staining signals were identified as red dots.

### Cell culture

NHLRC2 expression in stromal and epithelial cell lines were compared in vitro. Stromal cells were cultured from control lung tissue samples (n = 4) and surgical lung biopsy samples of the patients with IPF (n = 5) as described previously [18]. Briefly, the cells were cultured in medium consisting of Minimum essential medium Eagle α modification (Sigma-Aldrich) supplemented with 13% heat-inactivated fetal bovine serum (FBS-Good, Pan Biotech, Aidenbach, Germany), 2 mM l-glutamine, 100 U/ml penicillin, 0.1 g/l streptomycin, 2.5 mg/l amphotericin B and 10 mM HEPES (all from Sigma-Aldrich). These cell lines are composed of both fibroblasts and myofibroblasts as previously described in our electron microscopic analyses [18]. Cells were used for experiments in passages 3–6.

Normal human primary small airway epithelial cells (SAEC) and normal human primary bronchial/tracheal epithelial cells (PBTE) (American type culture collection, ATCC, Virginia, USA) were cultured in airway cell basal medium supplemented with bronchial epithelial growth kit (ATCC). SAEC were used for experiments in passages 6–7 and PBTE in passage 5.

In order to study the effect of TGF-β1 on NHLRC2 levels, one control and one IPF stromal cell line and SAEC were plated at a density of 9000 cells/cm^2^. After 24 h stromal cells were exposed to 5 ng/ml TGF-β1 (Sigma-Aldrich) in a serum-free growth medium for 24 to 72 h. SAECs were exposed to 5 ng/ml TGF-β1 in complete growth medium for 24 to 72 h. Cells cultured in similar conditions without TGF-β1 were used as controls.

### RNA extraction and RT-qPCR

Total RNA was extracted from cultured cells using the RNeasy Mini Kit (Qiagen, Hilden, Germany) according to the manufacturer’s instructions and the RNA concentrations were measured using the NanoDrop spectrophotometry system (Thermo Fisher Scientific, Vilnius, Lithuania). Five hundred-ng aliquots of RNA were reverse-transcribed using RevertAid First Strand cDNA Synthesis Kit (Thermo Fisher Scientific) using oligo(dT)_18_ as primer according to manufacturer’s instructions. PCR amplification was performed in triplicate as previously described [17, 18] by using iQTM SYBR Green Supermix (Bio-Rad Laboratories, Inc., Hercules, CA, USA). Primer sequences and annealing temperatures specific for each primer pair are listed in Additional file 1: Table S2. Relative gene expressions were quantified by using the 2^−∆∆C^T Livak method [19] and gene expression levels were normalized to glyceraldehyde 3-phosphate dehydrogenase (*GAPDH*). The normalized values were compared to average of the normal control stromal cell lines or non-treated samples in TGF-β1 experiments to calculate the fold changes.

### Immunoblotting

The cells were lysed in 1.5% dodecyl maltoside (DDM, in phosphate buffered saline) or in radio-immunoprecipitation assay (RIPA) lysis and extraction buffer (Thermo Fisher Scientific) supplemented with a protease inhibitor cocktail tablet (Roche, Mannheim, Germany). After 45-min incubation on ice the samples were centrifuged at 20,000 *g* for 20 min. The protein concentration of cell lysates was determined by DC Protein Assay Kit (Bio-Rad) according to the manufacturer’s instructions. Twenty-μg aliquots of samples were loaded with Bolt LDS sample buffer (Thermo Fisher Scientific) and run on SDS–PAGE (Invitrogen Bolt Bis–Tris Mini Protein Gels, Thermo Fisher Scientific). The proteins were transferred onto 0.45 µm nitrocellulose membrane (Optitran reinforced NC, Whatman Schleicher and Schuell, Dassel, Germany). After blocking with 5% skim milk, the membranes were incubated with primary antibodies for NHLRC2, α-SMA and GAPDH followed by appropriate labelled secondary antibody incubation. Details of antibodies are listed in Additional file 1: Table S1. Protein bands were visualised with an Odyssey infrared imager (LI-COR Biosciences, Lincoln, NE, USA) and quantified with Image Studio Lite (LI-COR Biosciences). The expression levels of the target proteins were normalized to that of GAPDH.

### Statistical analyses

IBM SPSS Statistics for Windows, Version 25.0 (IBM Corp, Armonk, NY) was used to perform statistical analysis. OriginPro, Version 2019b (OriginLab Corporation, Northampton, MA, USA) was used for preparing graphs. The data were presented as median values with 25 and 75% quartiles for skewed variables, or as the means with standard deviation for those with a normal distribution. Comparisons of parameters that were not normally distributed between more than two groups were performed using the Kruskal–Wallis test and post hoc analysis (Dunn’s test with Bonferroni correction) and between two groups using Mann–Whitney U test. FVC% and DLCO% were divided in two groups based on median values (75% and 53%, respectively) in IPF patients at a stable phase of the disease. Survival was evaluated using the Kaplan–Meier method and differences in survival curves were evaluated using the log-rank test. The associations of relative immunohistochemical NHLRC2 expression and the number of NHLRC2-positive FF/cm^2^ with survival or future AE were analysed using univariate Cox regression analysis. Median values of relative NHLRC2 expression and NHLRC2-positive FF/cm^2^ in IPF patients were used as cut-off values for Kaplan-Meyer and Cox regression analysis. Values of p < 0.05 were considered statistically significant.

## Results

The characteristics of the study subjects included in the immunohistochemical analysis are shown in Table 1. From the 50 IPF patients, 47 (94%) had surgical lung biopsy sample taken at the stable phase of the disease and 3 (6%) during AE, and 37 (76%) were male. Twenty-eight (62%) out of 45 patients with known smoking history were ever-smokers, including 8 current- and 20 ex-smokers.

**Table 1.** Characteristics of study subjects included in immunohistochemical analysis

Parameters	IPF (n = 50)	Control (n = 10)
*Tissue sample histology, n (%)*		
Normal	–	10 (100)
UIP	47 (94)	–
UIP and DAD	3 (6)	–
*Two separate lung tissue samples—Surgical lung biopsy and autopsy lung tissue specimen, n (%)*		
UIP in biopsy and UIP with DAD in autopsy	6 (12)	–
UIP with DAD in both biopsy and autopsy specimens	2 (4)	–
Age years, mean (SD)	62.3 (7.9)	70.5 (6.4)
*Gender, n (%)*		
Male	37 (74)	1 (10)
Female	13 (26)	9 (90)
*Smoking status, n (%)*^*a*^		
Never-smoker	17 (38)	9 (90)
Ex-smoker	20 (44)	1 (10)
Current smoker	8 (18)	0 (0)
Pack-years of ever-smokers, median (IQR)^b^	25.0 (19.5–37.0)	7
FVC%, mean (SD)^c^	73.8 (15.5)	100.8 (17.5)
FEV1%, mean (SD)^c^	78.4 (16.7)	99.3 (19.3)
DLCO%, median (IQR)^d^	53.0 (45.0–62.1)	87.8 (76.4–111.0)
Follow up time, months, median (IQR)^e^	41.3 (15.1–73.8)	–
Episode of AE during follow-up, n (%)	22 (44)	–
Diseased or transplanted, n (%)	39 (78)	–
Transplanted, n (%)	4 (8.0)	–

The values were from the time of surgical lung biopsy (IPF). For follow-up time, death or lung transplantation was used as an endpoint event. Follow-up time for patients having no endpoints was defined as the time between biopsy date and May 11, 2021. Non-smoking patients operated for lung cancer were used as controls

*AE* acute exacerbation, *DAD* diffuse alveolar damage, *DLCO*% percent predicted diffuse capacity for carbon monoxide, *FEV1*% percent predicted forced expiratory volume at one second, *FVC*% percent predicted forced vital capacity, *IQR* interquartile range, *n* number, *SD* standard deviation, *UIP* usual interstitial pneumonia

^a^IPF n = 45

^b^IPF n = 25

^c^IPF n = 42, control n = 9

^d^IPF n = 41, control n = 8

^e^IPF n = 49

Pharmacological treatments of IPF are presented in Table 2. The pharmacological treatment was started in most cases after the surgical lung biopsy operation, although one patient was treated with corticosteroid and one with corticosteroid combined with azathioprine before the biopsy. All patients with AE-IPF (n = 22) were treated with corticosteroids. Nine patients with either biopsy or autopsy samples taken at the time of AE-IPF received corticosteroid before operation. There were no differences in survival of patients who had received antifibrotic drug therapy, namely pirfenidone or nintedanib, (n = 18, median 75.1 months, 95% confidence interval 39.7–110.5) and others (n = 28, median 40.6, 95% confidence interval 24.2–57.1, Log Rank p = 0.430) in the current study population.

**Table 2:** Pharmacological treatment of idiopathic pulmonary fibrosis

Medication^a^	n (%)
No medication^b^	9 (18)
Corticosteroids	25 (50)
Azathioprine	12 (24)
N-acetylcysteine	5 (10)
Cyclophosphamide	5 (10)
Triple therapy^c^	11 (22)
Pirfenidone	16 (32)
Nintedanib	5 (10)

^a^23 patients received several therapeutic treatments at different times

^b^Including 3 patients whose surgical lung biopsy was taken at the time of acute exacerbation of idiopathic pulmonary fibrosis

^c^Triple therapy = azathioprine, N-acetylcysteine and prednisolone

### Cell type-specific NHLRC2 protein and mRNA expression in IPF and controls

**Fig. 1:**
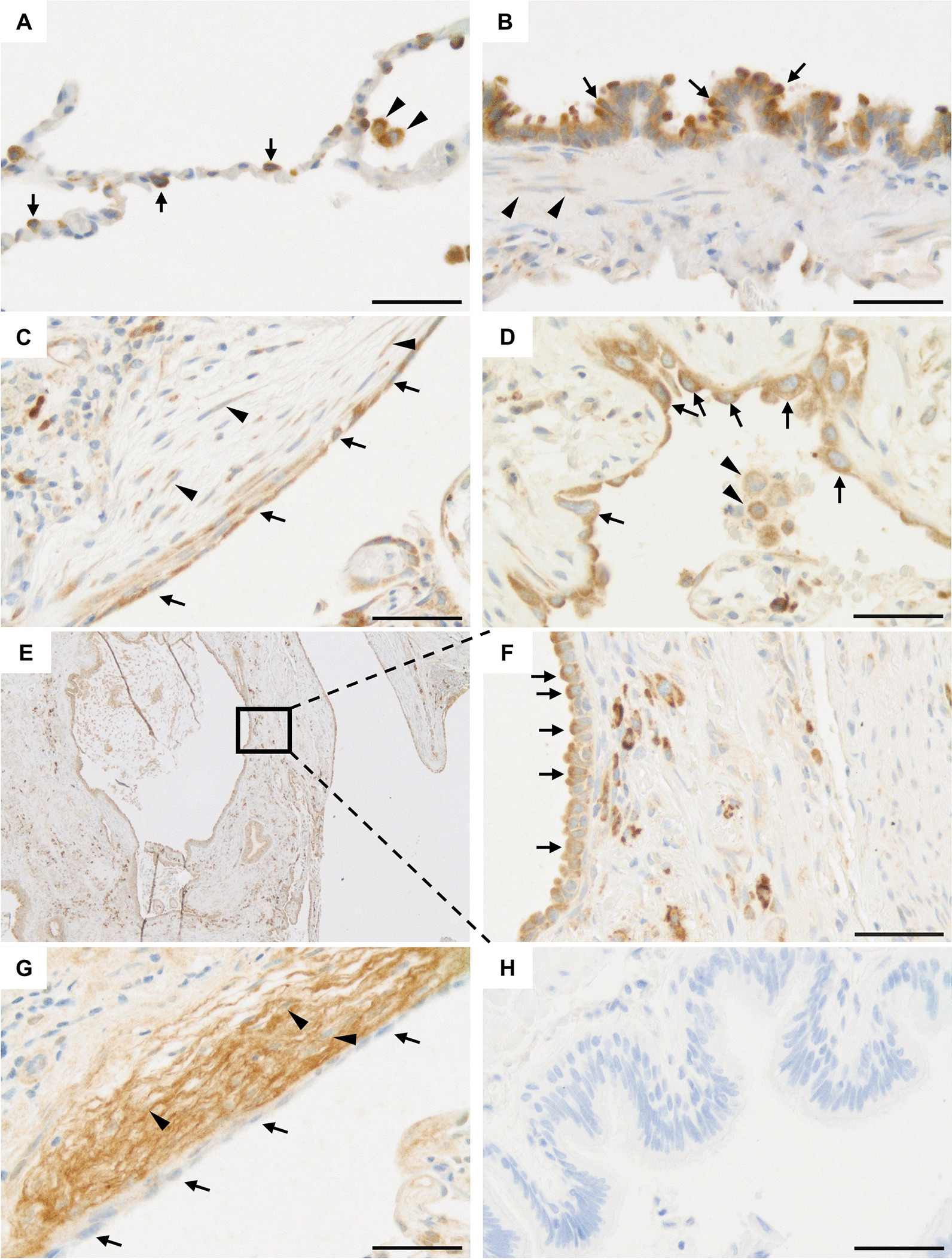
Immunohistochemical NHLRC2 and collagen α1(IV) chain expression in control lung and idiopathic pulmonary fibrosis (IPF). **A** A strong cytoplasmic NHLRC2 expression in type II pneumocytes (arrows) and alveolar macrophages (arrowheads) in a control lung. **B** Normal small airway epithelial cells (arrows) were positive for NHLRC2 while smooth muscle cells (arrowheads) were weakly positive or negative. **C** In lung tissues of IPF patients, hyperplastic alveolar epithelial cells (arrows) lining fibroblast focus, and some stromal cells (arrowheads) were positive for NHLRC2. **D** Hyperplastic epithelial cells (arrows) and alveolar macrophages (arrowheads) were positive for NHLRC2 in IPF. **E**, **F** Hyperplastic alveolar epithelial cells (arrows) lining honeycombs were strongly positive for NHLRC2. **G** Stromal cells (arrowheads) of a fibroblast focus were positive for collagen α1(IV) chain, while hyperplastic alveolar epithelial cells (arrows) lining the fibroblast focus were weak or negative in IPF. **H** Negative control in which primary antibody was substituted with rabbit isotype control. Scale bar 50 µm

The cell-type specific NHLRC2 protein and mRNA expression in normal lung and IPF was studied by IHC and in situ hybridization. In normal lung, strong cytoplasmic immunohistochemical NHLRC2 expression was detected in alveolar type II pneumocytes, small airway epithelial cells and alveolar macrophages (Fig. 1A, B). From negative to strong NHLRC2 expression was detected in endothelial cells. Weak NHLRC2 immunoreactivity was occasionally observed in type I pneumocytes and smooth muscle cells. In IPF, at the stable phase and during AE, mainly moderate to strong cytoplasmic NHLRC2 expression was observed in hyperplastic/metaplastic alveolar and bronchiolar type epithelial cells lining FF and honeycombs (Fig. 1C–F). Some spindle shaped stromal cells within FF (positive for α-SMA) were also positive for NHLRC2 (Fig. 1C) in IPF. The expression of collagen α1(IV) was different compared to that of NHLRC2 since it was mainly observed extracellularly within the stromal cells of FF, while a very weak immunoreactivity was observed in the hyperplastic alveolar epithelial cells lining FF and honeycombs (Fig. 1G).

Although the autopsy lung tissue samples of IPF patients contained varying amounts of autolysis, mainly moderate to strong NHLRC2 expression was observed in hyperplastic alveolar and bronchiolar epithelial cells similar to lung biopsy samples.

**Fig. 2:**
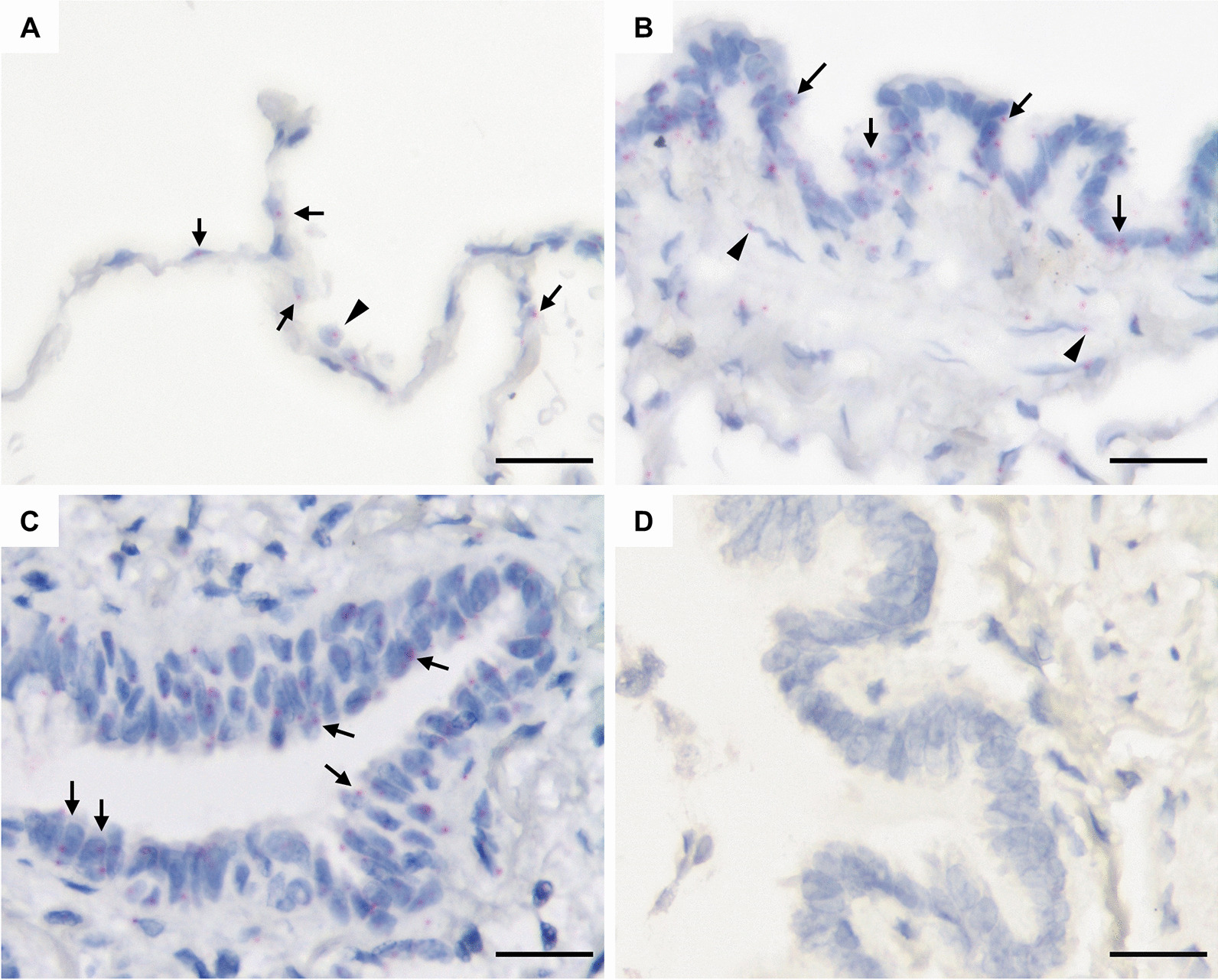
*NHLRC2* mRNA expression in a control lung and idiopathic pulmonary fibrosis (IPF) by in situ hybridization. **A** *NHLRC2* expression in normal alveolar epithelial cells (arrows), and alveolar macrophage (arrowhead). **B** Small airway epithelial cells (arrows) and some smooth muscle cells (arrowheads) express *NHLRC2*. **C** *NHLRC2* expression in hyperplastic alveolar epithelial cells in IPF. **D** Negative control for mRNA in situ hybridization. Scale bar 25 µm

*NHLRC2* mRNA expression was in line with protein expression being observed in alveolar epithelial cells and bronchiolar epithelial cells (Fig. 2A, B) in normal lung. *NHLRC2* expression was also detected in some alveolar macrophages, smooth muscle cells and endothelial cells. In IPF *NHLRC2* expression was observed in hyperplastic alveolar epithelial cells lining FF and honeycombs (Fig. 2C). Some individual stromal cells within FF also expressed *NHLRC2*.

### Immunohistochemical NHLRC2 expression in IPF compared to control lung

**Fig. 3:**
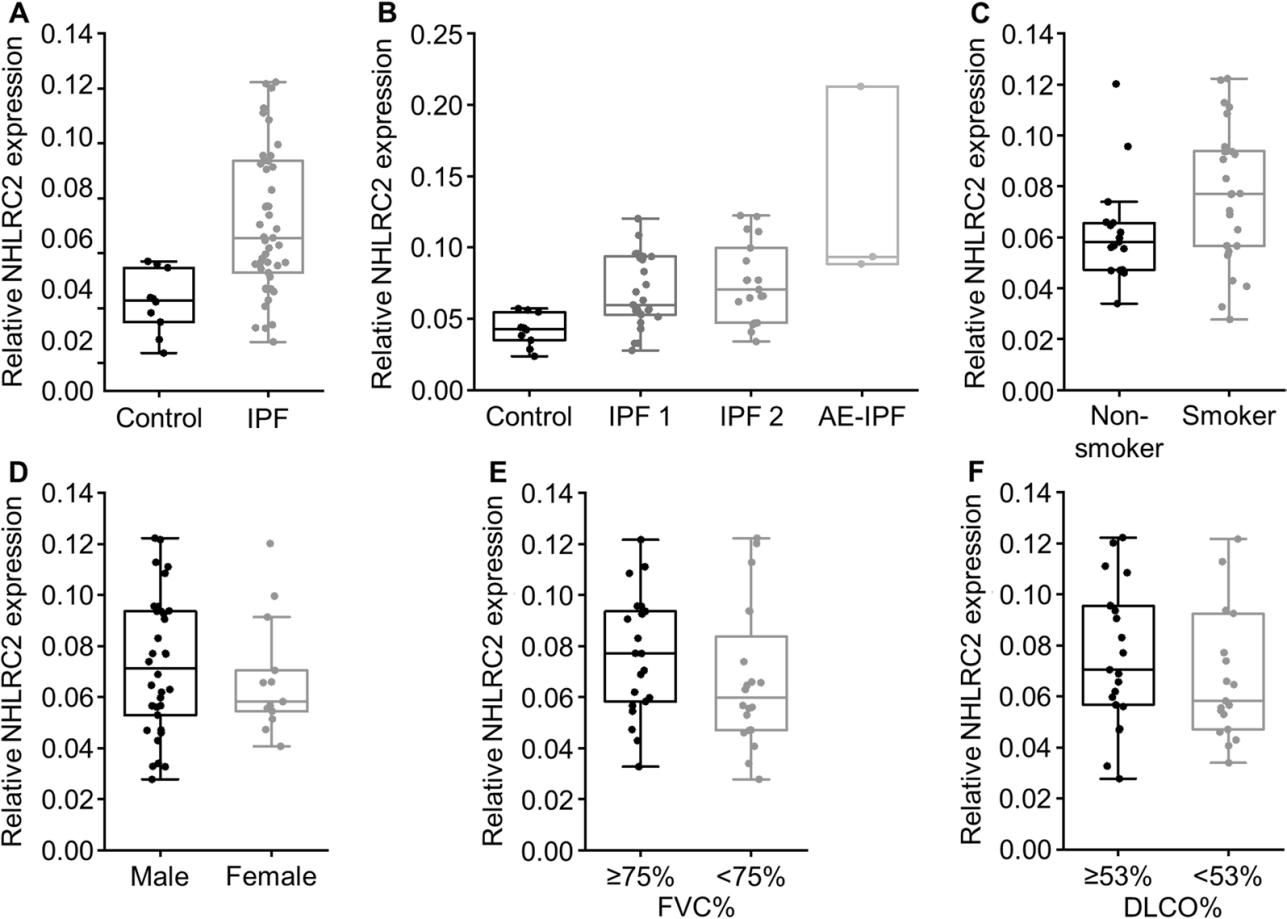
Relative NHLRC2 protein expression in idiopathic pulmonary fibrosis (IPF) and control lungs. The area of positive immunohistochemical NHLRC2 expression was quantified with Visiopharm digital pathology image analysis software in relation to the whole tissue section area. **A** NHLRC2 expression was higher in IPF patients at the stable phase of the disease (n = 47) compared to controls (n = 10) (p < 0.001). **B** NHLRC2 expression was higher in IPF patients not experiencing acute exacerbation (AE) during follow-up (IPF 1, n = 28), IPF patients experiencing AE during follow-up (IPF 2, n = 19), and IPF patients experiencing AE at the time of biopsy (AE-IPF, n = 3) compared to controls (n = 10) (p = 0.022, p = 0.005, p = 0.007, respectively). There was a trend of AE-IPF patients (n = 3) having a higher NHLRC2 expression compared to IPF patients at the stable phase of the disease not experiencing AE during follow-up (IPF 1, n = 28), and patients experiencing AE during follow-up (IPF 2, n = 19) (p = 0.473 and p = 1.000, respectively). **C** There was a trend of current and ex-smokers (n = 26) having higher NHLRC2 expression compared to non-smokers (n = 17) (p = 0.060) at the stable phase of the disease. **D**–**F** NHLRC2 expression did not differ between males (n = 34) and females (n = 13) (p = 0.549), in patients IPF patients with high percent predicted forced vital capacity (FVC%) (n = 21) and low FVC% (n = 20) (p = 0.190) or in patients with high percent predicted diffusing capacity for carbon monoxide (DLCO%) (n = 21) and low DLCO% (n = 19) (p = 0.247)

Immunohistochemical NHLRC2 expression in IPF and control lung tissue samples was compared with digital pathology image analysis. The relative NHLRC2-positive area was higher in IPF at a stable phase of the disease (n = 47) compared to control (n = 10) (p < 0.001, Fig. 3A). There was a trend of AE-IPF patients (n = 3, median 0.0935, IQR 0.0910–0.1532) having a higher NHLRC2 expression than the patients in the stable phase of the disease (n = 47, median = 0.0656, IQR = 0.0537–0.0931) (p = 0.080), although the difference was not statistically significant. There were no differences between IPF patients experiencing AE during follow-up (n = 19) and IPF patients who did not (n = 28) (p = 1.000) at the stable phase of the disease (Fig. 3B) in NHLRC2 expression. At the stable phase of the disease, ever-smokers (n = 26) seemed to have higher relative NHLRC2 expression compared to non-smokers (n = 17) (p = 0.060) (Fig. 3C), although the difference was not statistically significant. However, when the two AE-IPF patients with known smoking history were included in the analysis, ever-smokers (n = 28, median = 0.0801, IQR = 0.0566–0.0937) had significantly higher NHLRC2 expression than non-smokers (n = 17, median = 0.0582, IQR = 0.0472–0.0656) (p = 0.037). There were no differences in relative NHLRC2 expression between females and males high and low FVC% or high and low DLCO% (Fig. 3D–F).

Kaplan–Meier survival analysis did not show differences in survival between low (< 0.0674) and high (≥ 0.0674) relative NHLRC2 expressions. Immunohistochemical NHLRC2 expression in tissue samples obtained at the stable phase of IPF did not predict future episode of AE or survival.

### NHLRC2 expression in stromal cells in fibroblast foci

The numbers of NHLRC2-positive FF (more than 50% of spindle-shaped cells within FF were positive) were calculated to study the NHLRC2 expression especially in fibroblasts and myofibroblasts. We did not observe differences in the numbers of NHLRC2-positive FF/cm^2^ between IPF patients at the stable phase of the disease experiencing AE during follow-up time (n = 19) and patients who did not (n = 28) (p = 0.485), females (n = 13) and males (n = 34) (p = 0.581), ever-smokers (n = 26) and non-smokers (n = 17) (p = 0.453), high (n = 21) and low FVC% (n = 20) (p = 0.579) or high (n = 21) and low DLCO% (n = 19) (p = 0.728). There were no differences in patient survival in low (< 10.3 FF/cm^2^) and high (≥ 10.3 FF/cm^2^) number of NHLRC2-positive FF. The number of NHLRC2-positive FF/cm^2^ did not predict future episode of AE-IPF or survival.

**Fig. 4:**
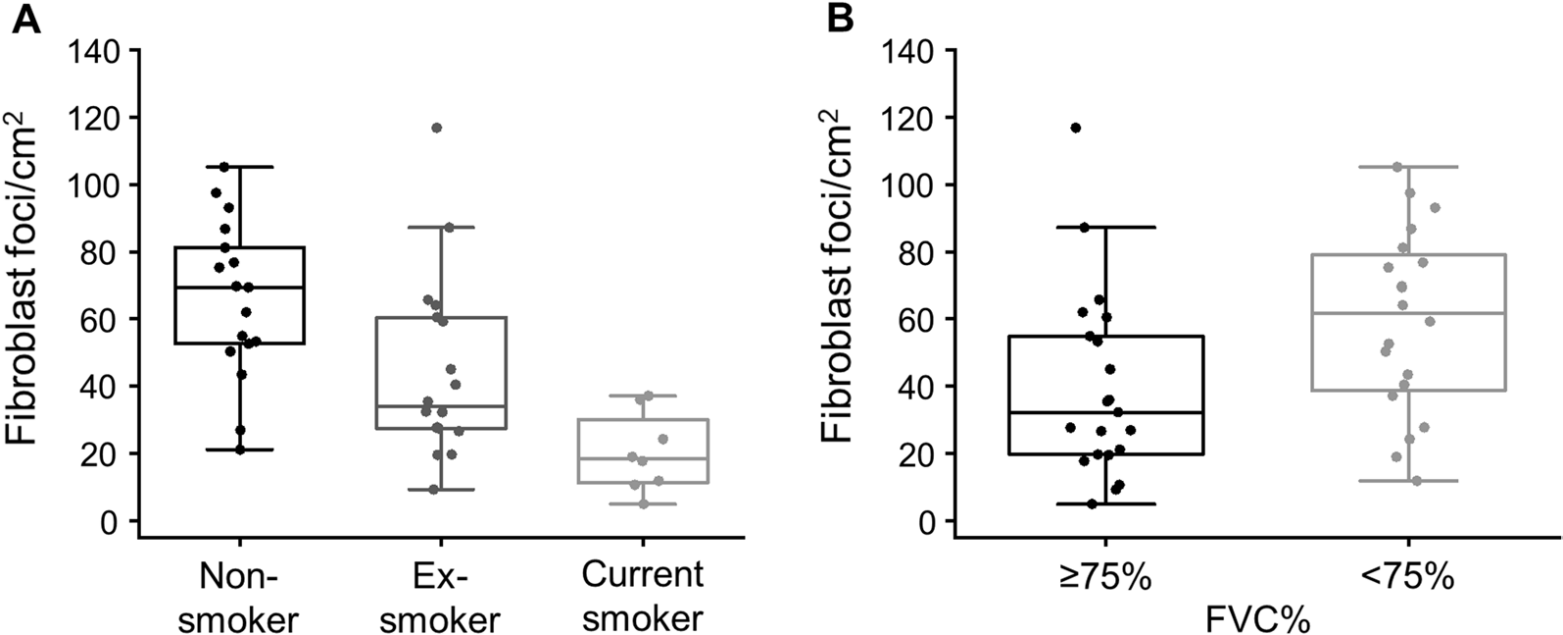
The number of fibroblast foci (FF) in idiopathic pulmonary fibrosis (IPF). The total number of FF were calculated from tissue sections of IPF patients (n = 47) with UIP histology in relation to the tissue section area. **A** Current smokers (n = 8) had less FF/cm^2^ compared to non-smokers (n = 17) (p < 0.001). There was a trend of ex-smokers (n = 18) having less FF/cm^2^ compared to non-smokers (n = 17) (p = 0.068) and current smokers having less FF/cm^2^ compared to ex-smokers (p = 0.078). **B** IPF patients with low percent predicted forced vital capacity (FVC%) (n = 20) had more FF/cm^2^ compared to patients who had high FVC% (n = 21) (p = 0.020)

In addition to the NHLRC2-positive FF, the total number of FF was counted. The number of FF/cm^2^ was lower in ever-smokers (n = 26, median = 29.9, IQR = 19.5–45.0 FF/cm^2^) than in non-smokers (n = 17, median = 69.4, IQR = 52.6–81.2 FF/cm^2^) (p < 0.001). There was a trend of current smokers (n = 8) having less FF/cm^2^ than ex-smokers (n = 18) (p = 0.068, Fig. 4A). Additionally, patients with low FVC% (n = 20) had more FF/cm^2^ than patients with higher FVC% (n = 21) (p = 0.020, Fig. 4B). There were no differences in the numbers of FF/cm^2^ in patients experiencing AE-IPF during follow-up (n = 19) compared to patients not experiencing AE-IPF (n = 28) (p = 0.540), males (n = 34) and females (n = 13) (p = 0.751) or high (n = 21) and low DLCO% (n = 19) (p = 0.436).

### NHLRC2 mRNA and protein levels in cultured lung cell lines

**Fig. 5:**
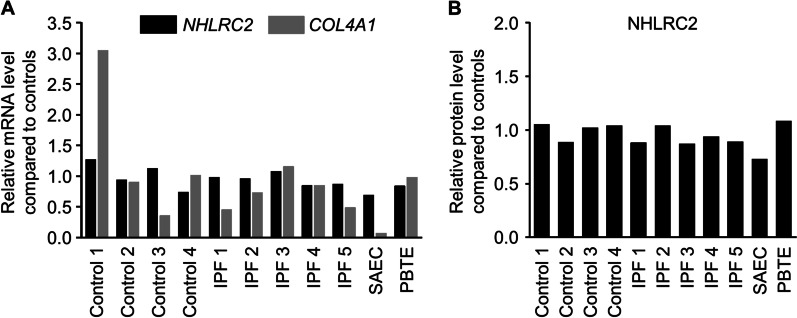
NHLRC2 mRNA and protein levels in stromal and epithelial cell lines. Total RNA was isolated and cellular lysates were prepared from stromal cells cultured from control lungs (n = 4) and idiopathic pulmonary fibrosis (IPF) patients (n = 5), small airway epithelial cells (SAEC) (n = 1), and primary bronchial/tracheal epithelial cells (PBTE) (n = 1). **A** *NHLRC2* and *COL4A1* levels were measured with quantitative reverse transcription polymerase chain reaction, normalized to *GAPDH*, and represented graphically as the fold change compared to average of control cell lines. **B** NHLRC2 band intensities were quantified from immunoblots (shown in Additional file 2: Figure S1A) using Image Studio Lite software, normalized to GAPDH, and compared to average of the control cell lines

NHLRC2 mRNA and protein levels were measured in different types of cultured cells with RT-qPCR and Western blot analysis, respectively, to evaluate whether the expression differs between stromal cells and epithelial cells. In the cell culture conditions NHLRC2 mRNA and protein levels in cultured stromal cells were similar compared to SAEC and PBTE (Fig. 5A, B). *COL4A1* expression, instead, was lower in SAEC compared to stromal cell lines (Fig. 5A).

### NHLRC2 expression was not regulated by TGF-β1 in vitro

**Fig. 6:**
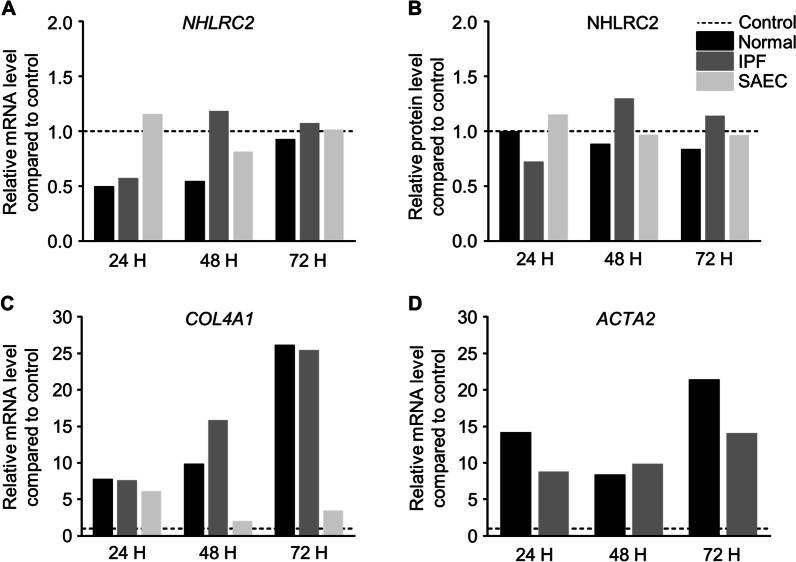
The effect of transforming growth factor (TGF)-β1 on NHLRC2 expression. Primary stromal cells derived from normal control lung (n = 1) and idiopathic pulmonary fibrosis (IPF) (n = 1), and small airway epithelial cell line (SAEC, n = 1) were exposed to 5 ng/ml TGF-β1 for 24 to 72 h. Total RNA was isolated and cellular lysates were prepared. **A** *NHLRC2* levels were measured by reverse transcriptase polymerase chain reaction (RT-qPCR), normalized to *GAPDH*, and represented graphically as the fold change compared to non-treated cells (control). **B** NHLRC2 band intensities were quantified from immunoblots (shown in Additional file 2: Figure S1B) using Image Studio Lite software, normalized to GAPDH, and compared to non-treated cells. **C**, **D** *COL4A1* and *ACTA2* levels were measured by RT-qPCR, normalized to *GAPDH,* and represented graphically as the fold change compared to non-treated cells (control) to confirm the fibroblast to myofibroblast activation by TGF-β1

To study whether TGF-β1 regulates NHLRC2 mRNA or protein expression in either stomal cells or epithelial cells, cultured stomal cells and SAEC were exposed to 5 ng/ml TGF-β1 for 24 to 72 h. TGF-β1 exposure did not have effect on NHLRC2 mRNA or protein levels in stromal cells or SAEC (Fig. 6A, B) in cell culture conditions. *COL4A1* and α-SMA mRNA and protein levels were measured to confirm the fibroblast to myofibroblast activation by TGF-β1. *COL4A1* and *ACTA2* levels were higher in TGF-β1 treated samples than in non-treated samples in stromal cells and SAEC (Fig. 6C, D). Also, α-SMA protein levels were higher in TGF-β1 treated samples compared to non-treated samples (Additional file 2: Figure S1C).

## Discussion

To our knowledge, this is the first study describing localization of NHLRC2 in IPF and control lung tissues. NHLRC2 was expressed in type II pneumocytes, alveolar macrophages and bronchiolar epithelial cells in control lung. In the lung tissue of the IPF patients, NHLRC2 was mainly expressed in hyperplastic alveolar and bronchial epithelial cells lining FF and honeycombs. We found that immunohistochemical NHLRC2 expression was higher in IPF compared to control lung and in ever-smokers compared to non-smokers. TGF-β1 did not have effect on NHLRC2 mRNA or protein expression in primary lung stromal or epithelial cell lines in vitro.

NHLRC2 contains thioredoxin (TRX)-like domain, although it has not been shown to have thioredoxin activity so far [10, 20]. NHLRC2 expression in lung observed in this study resembles that of TRX, since in the previous study TRX1 was expressed in the hyperplastic alveolar epithelium, bronchiolar epithelial cells, and alveolar macrophages in IPF [21, 22]. TRX1 levels in serum has been shown to be higher in IPF compared to control and in patients experiencing AE later compared to patients not having AE [22]. The function of NHLRC2 is not fully understood yet. It has been shown to be involved in the regulation of phagocytosis in macrophages in two genome-wide knockout screens [20, 23] and it has been suggested to have a role in the regulation of ROS-induced apoptosis [24].

Smoking has been found to increase the risk of having IPF in several studies [25, 26]. Additionally, current smokers have been shown to have longer unadjusted survival time compared to non- and ex-smokers [27, 28]. However, smoking status has been rarely compared with immunohistochemical observations. In the current study, we observed that the immunohistochemical NHLRC2 expression was higher in ever-smokers compared to non-smokers and, and moreover, that ever-smokers had less FF compared to non-smokers. In contrast, current smokers were shown to have less mast cells compared to non- or ex-smokers in our previous study [16] and thus, it could be useful to compare smoking history in addition to clinical parameters to immunohistochemical data since it could reveal novel information of disease pathogenesis.

*NHLRC2* was listed as down-regulated gene in lung tissues of IPF patients whose FVC% and DLCO% values declined significantly up to 12 months following lung biopsy compared to slowly progressing disease [13]. In the current study, however, the study protocol was different since we analysed the pulmonary function test results at the time of biopsy and information of the patients experiencing AE-IPF during follow-up time. We did not detect associations in FVC%, DLCO% or occurrence of AE-IPF and NHLRC2 expression. However, three patients having AE-IPF at the time of biopsy seemed to have higher NHLRC2 expression than patients in stable phase of the disease.

TGF-β1 induces fibroblast to myofibroblast differentiation, extracellular matrix production, and epithelial to mesenchymal transition in lung epithelial cells [29–31]. Variants of *NHLRC2* have been shown to enhance fibroblast to myofibroblast differentiation in skin fibroblasts derived from patients with fibrosis, neurodegeneration, and cerebral angiomatosis disease [32]. The effect of TGF-β1 on NHLRC2 expression has not been investigated before. In the current study, TGF-β1 induction did not reveal an effect on NHLRC2 mRNA or protein level either in primary lung stromal cells or SAEC. Similarly, TGF-β1 did not alter the protein levels of peroxiredoxins in two human epithelial lung cell lines (A549 and BEAS-2B) [33]. Stimulation with TGF-β1 has been shown to result in the upregulation of *COL4A1* in lung and renal fibroblasts [34, 35] which is in line with our observation of higher *COL4A1* mRNA levels in TGF-β1 treated lung cells compared to non-treated cells.

This study had some limitations as it was a retrospective investigation with a limited number of patients which leads to reduced statistical power. However, in comparison to other studies that have used histological material from IPF patients, we have quite high number of samples [36]. Furthermore, the patients were treated with various pharmacological therapies for IPF, which may have affected the occurrence of AE-IPF and survival.

## Conclusions

NHLRC2 expression was higher in IPF compared to controls being widely expressed in type II pneumocytes, macrophages, bronchiolar epithelium, and hyperplastic alveolar epithelium and its expression was associated with smoking. Additionally, its expression was not regulated by the exposure to TGF-β1 in vitro. Further studies are needed to clarify the role of NHLRC2 in IPF.

## Supplementary Information

**Additional file 1: Table S1.** Antibodies used for immunohistochemistry and Western blot analysis. **Table S2.** Sequences, annealing temperatures, and amplicon sizes of primers used for RT-qPCR.

**Additional file 2: Figure S1.** Immunoblots of NHLRC2 and α-SMA expression in cultured lung cells. (A) Immunoblot of NHLRC2 expression in cell lysates prepared from primary stromal cells derived from control lungs (n = 4) and idiopathic pulmonary fibrosis (IPF) patients (n=5), small airway epithelial cells (SAEC) (n = 1), and primary bronchial/tracheal epithelial cells (PBTE) (n = 1). (B, C) Primary stromal cells derived from normal control lung (n = 1) and IPF (n = 1), and small airway epithelial cell line (SAEC, n = 1) were exposed to 5 ng/ml TGF-β1 for 24 to 72 hours, cell lysates were prepared and subjected to SDS-PAGE and Western blot analysis. (B) Immunoblot of NHLRC2 expression in TGF-β1 exposed cells. (C) Immunoblot and a bar graph of alpha smooth muscle actin (α-SMA) levels. Band intensities were quantified using Image Studio Lite software, normalized to GAPDH, and compared to non-treated cells (control)

## Data Availability

The data generated during the current study are available from the corresponding author on a reasonable request.

## Abbreviations

α-SMA, ACTA2: Alpha smooth muscle actin
AE-IPF: Acute exacerbation of idiopathic pulmonary fibrosis
COL4A1: Collagen alpha 1(IV) chain
DAD: Diffuse alveolar damage
DLCO%: Percent predicted diffusing capacity of carbon monoxide
FF: Fibroblast focus
FVC%: Percent predicted forced vital capacity
GAPDH: Glyceraldehyde 3-phosphate dehydrogenase
IHC: Immunohistochemistry
IQR: Inter quartile range
IPF: Idiopathic pulmonary fibrosis
NHLRC2: NHL-repeat containing protein 2
PBTE: Primary bronchial/tracheal epithelial cells
SAEC: Small airway epithelial cells
TGF-β1: Transforming growth factor beta 1
TRX: Thioredoxin
UIP: Usual interstitial pneumonia
RT-qPCR: Quantitative reverse transcription polymerase chain reaction
