# Supplementary material for: NHLRC2 expression is increased in idiopathic pulmonary fibrosis
Source: Respir Res. 2022 Aug 13;23:206. doi: 10.1186/s12931-022-02129-z (PMC9375339; doi:10.1186/s12931-022-02129-z)
Supplement: Supplementary file 1 — Additional file 1: Table S1. Antibodies used for immunohistochemistry and Western blot analysis. Table S2. Sequences, annealing temperatures, and amplicon sizes of primers used for RT-qPCR. [file 12931_2022_2129_MOESM1_ESM.pdf]

Table S1. Antibodies used for immunohistochemistry and Western blot analysis.

Antibody	Supplier and catalogue number	Epitope retrieval in IHC	Dilution		Incubation
			IHC	WB	
**Primary antibody**					
Polyclonal rabbit anti-NHLRC2	Sigma-Aldrich, HPA038493	MW in Tris-EDTA buffer (pH 9) for 15 min	1:500	1:500 in TBS-T	30 min at RT and o/n +4°C (IHC), o/n +4°C (WB)
Polyclonal rabbit anti-collagen IV alpha 1	Novus Biologicals (Abingdon, UK), NB120-6586	Pepsin treatment for 30 min at 37°C	1:75	-	1h at RT
Monoclonal mouse smooth muscle actin (clone 1A4)	Dako (Glostrup, Denmark), M0851	MW in Tris-EDTA buffer (pH 9) for 15 min	1:1,000	1:1,000 in TBS-T	30 min at RT (IHC), o/n at +4°C (WB)
Monoclonal mouse anti-human CD68 (clone PG-M1)	Dako, M0876	MW in Tris-EDTA buffer (pH 9) for 15 min	1:300	-	30 min at RT
Monoclonal mouse anti-thyroid transcription factor (clone 8G7G3/1)	Dako, M3575	MW in Tris-EDTA buffer (pH 9) for 15 min	1:200	-	30 min at RT
Monoclonal mouse anti-human CD31 (clone JC70A)	Dako, M0823	MW in Tris-EDTA buffer (pH 9) for 15 min	1:100	-	30 min at RT

Rabbit polyclonal anti-GAPDH	Abcam (Cambridge, UK), ab9485	-		1:2,500 in TBS-T	o/n at +4°C
Mouse monoclonal anti-GAPDH (clone C65)	Abcam, ab8245	-		1:5,000 in TBS-T	o/n at +4°C
**Secondary antibody**					
Donkey anti-mouse IRDye680RD	LI-COR Biosciences (Lincoln, NE, USA), 925-68072	-	-	1:10,000 in TBS-T	1h at RT
Donkey anti-mouse IRDye800CW	LI-COR, 926-32212	-	-	1:10,000 in TBS-T	1h at RT
Donkey anti-rabbit IRDye800CW	LI-COR, 925-32213	-	-	1:10,000 in TBS-T	1h at RT
Donkey anti-rabbit IRDye700DX	Rockland Immunochemicals (Pottstown, PA, USA), 611-730-127	-	-	1:5,000 in TBS-T	1h at RT

IHC, immunohistochemistry; MW, microwave heat treatment; o/n, overnight; RT, room temperature; TBS-T, tris-buffered saline supplemented with 0.1% tween-20; WB, Western blot analysis.

Table S2. **Sequences, annealing temperatures, and amplicon sizes of primers used for RT-qPCR.**

Target	Primer	Sequence 5'-3'	Ta (°C)	Amplicon size (bp)
ACTA2	Forward	GCAGCCCAGCCAAGCACTGT	63	135
	Reverse	TGGGAGCATCGTCCCCAGCA		
COL4A1	Forward	CCCCAAAGGTGTTGACGGCT	61.4	126
	Reverse	AGACCAACTCCAGGCTCTCC		
GAPDH	Forward	GAGTCAACGGATTTGGTCGT	63	185
	Reverse	GACAAGCTTCCCGTTCTCAG		
NHLRC2	Forward	AGCTGAAGGCAATGAATGGCTACT	59	173
	Reverse	TACAAGCACTGCTGTCTGCACTA		
