# Supplementary material for: NHLRC2 expression is increased in idiopathic pulmonary fibrosis
Source: Respir Res. 2022 Aug 13;23:206. doi: 10.1186/s12931-022-02129-z (PMC9375339; doi:10.1186/s12931-022-02129-z)
Supplement: Supplementary file 2 — Additional file 2: Figure S1. Immunoblots of NHLRC2 and α-SMA expression in cultured lung cells. (A) Immunoblot of NHLRC2 expression in cell lysates prepared from primary stromal cells derived from control lungs (n = 4) and idiopathic pulmonary fibrosis (IPF) patients (n=5), small airway epithelial cells (SAEC) (n = 1), and primary bronchial/tracheal epithelial cells (PBTE) (n = 1). (B, C) Primary stromal cells derived from normal control lung (n = 1) and IPF (n = 1), and small airway epithelial cell line (SAEC, n = 1) were exposed to 5 ng/ml TGF-β1 for 24 to 72 hours, cell lysates were prepared and subjected to SDS-PAGE and Western blot analysis. (B) Immunoblot of NHLRC2 expression in TGF-β1 exposed cells. (C) Immunoblot and a bar graph of alpha smooth muscle actin (α-SMA) levels. Band intensities were quantified using Image Studio Lite software, normalized to GAPDH, and compared to non-treated cells (control) [file 12931_2022_2129_MOESM2_ESM.pdf]

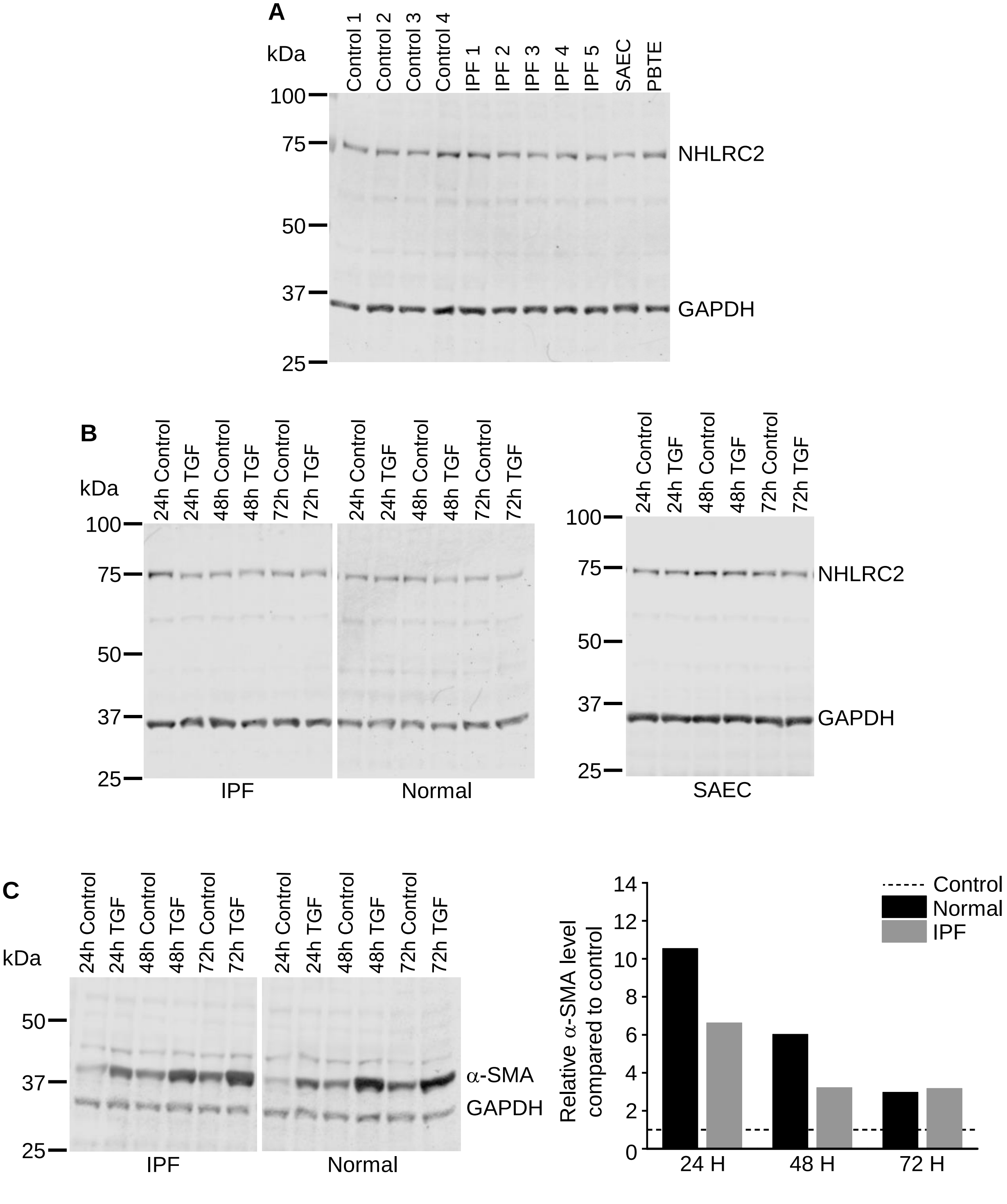
A
kDa
Control 1
Control 2
Control 3
Control 4
IPF 1
IPF 2
IPF 3
IPF 4
IPF 5
SAEC
PBTE
100
75
50
37
25
NHLRC2
GAPDH
B
kDa
24h Control
24h TGF
48h Control
48h TGF
72h Control
72h TGF
100
75
50
37
25
NHLRC2
GAPDH
IPF
Normal
SAEC
C
kDa
50
37
25
α-SMA
GAPDH
IPF
Normal
Relative α-SMA level compared to control
14
12
10
8
6
4
2
0
Control
Normal
IPF
24 H
48 H
72 H

Figure S1. Immunoblots of NHLRC2 and α-SMA expression in cultured lung cells. (A) Immunoblot of NHLRC2 expression in cell lysates prepared from primary stromal cells derived from control lungs (n = 4) and idiopathic pulmonary fibrosis (IPF) patients (n=5), small airway epithelial cells (SAEC) (n = 1), and primary bronchial/tracheal epithelial cells (PBTE) (n = 1). (B, C) Primary stromal cells derived from normal control lung (n = 1) and IPF (n = 1), and small airway epithelial cell line (SAEC, n = 1) were exposed to 5 ng/ml TGF-β1 for 24 to 72 hours, cell lysates were prepared and subjected to SDS-PAGE and Western blot analysis. (B) Immunoblot of NHLRC2 expression in TGF-β1 exposed cells. (C) Immunoblot and a bar graph of alpha smooth muscle actin (α-SMA) levels. Band intensities were quantified using Image Studio Lite software, normalized to GAPDH, and compared to non-treated cells (control).
